# High-Uniform and High-Efficient Color Conversion Nanoporous GaN-Based Micro-LED Display with Embedded Quantum Dots

**DOI:** 10.3390/nano11102696

**Published:** 2021-10-13

**Authors:** Yu-Ming Huang, Jo-Hsiang Chen, Yu-Hau Liou, Konthoujam James Singh, Wei-Cheng Tsai, Jung Han, Chun-Jung Lin, Tsung-Sheng Kao, Chien-Chung Lin, Shih-Chen Chen, Hao-Chung Kuo

**Affiliations:** 1 Department of Photonics, Institute of Electro-Optical Engineering, National Yang Ming Chiao Tung University, Hsinchu 30010, Taiwan; s101328035@gmail.com (Y.-M.H.); kingko56@yahoo.com.tw (J.-H.C.); randyliouyuhau@gmail.com (Y.-H.L.); jamesk231996@gmail.com (K.J.S.); jj510382.ee10@nycu.edu.tw (W.-C.T.); 18618135355@flexpn.com (C.-J.L.); tskao@nycu.edu.tw (T.-S.K.); 2 Institute of Photonic System, National Yang Ming Chiao Tung University, Tainan 71150, Taiwan; 3 Semiconductor Research Center, Hon Hai Research Institute, Taipei 11492, Taiwan; 4 Department of Electrical Engineering, Yale University, New Haven, CT 06520, USA; jung.han@yale.edu; 5 Graduate Institute of Photonics and Optoelectronics, National Taiwan University, Taipei 10617, Taiwan

**Keywords:** micro-LED, quantum dot, nanoporous-GaN, high uniform

## Abstract

Quantum dot (QD)-based RGB micro-LED technology is seen as one of the most promising approaches towards full color micro-LED displays. In this work, we present a novel nanoporous GaN (NP-GaN) structure that can scatter light and host QDs, as well as a new type of micro-LED array based on an NP-GaN embedded with QDs. Compared to typical QD films, this structure can significantly enhance the light absorption and stability of QDs. As a result, the green and red QDs exhibited light conversion efficiencies of 90.3% and 96.1% respectively, leading to improvements to the luminous uniformity of the green and red subpixels by 90.7% and 91.2% respectively. This study provides a viable pathway to develop high-uniform and high-efficient color conversion micro-LED displays.

## 1. Introduction

Since the last few years, the evolution of micro-light emitting diodes (μ-LED) has been accelerating, with numerous research advances, and industrial demand appearing one after another, and hence the number of researchers working on μ-LED displays has steadily increased [1]. Micro-LED display technology outperforms TFT-LCD and OLED displays in terms of brightness, efficiency, high transparency, high resolution, high contrast, low energy consumption, high response time, high viewing angle, image definition, and improved lifetime [2,3]. Micro-LEDs are primed to be the next breakthrough technology in the industry as demand for greater and higher resolution displays continues to grow [4,5]. In recent years, only micro-LED has been able to satisfy the high-resolution requirements of popular near-eye displays such as virtual reality (VR) and augment reality (AR) by offering high brightness [6,7], enhanced robustness, longer lifetimes and smaller form factors. In addition, micro-LEDs are also game-changing technology for a variety of different applications [8,9], ranging from large-area displays and televisions to mobile phones, smartwatches, automotive panels, visible light communication (VLC) [10,11], and flexible lighting. However, for micro-LEDs with a size below 80 μm, the substrate must be removed to minimize the overall dimensions. Furthermore, micro-LED faces a significant hurdle in the form of mass transfer technology [12], which is an obstacle to the commercialization of micro-LED displays. Mass transfer technology is a fundamental stage for the integration of micro-LED chips from the fabrication wafers onto the display panels. However, when it comes to RGB displays, the materials and structure of three-color LED chips are different in operating conditions. For example, a total of 24.9 million micro-LED dies are required to assemble a 4K micro LED screen, assuming that each sub-pixel is a die. It is not only time-consuming and low-yielding, but it is also a serious impediment to inspection and repair. Despite numerous attempts to design a cost-effective mass transfer method, solutions have yet to reach commercialization standards in terms of manufacturing output (in units per hour, UPH), transfer yield, and LED chip size. Epitaxy homogeneity, varied material operation circumstances, detection, and repair are the key challenges posed by mass transfer. Multiple RGB LED chips are transferred to a backplane using mass transfer technology, which incorporates a pick-and-place method [13,14], enabling large-scale micro-LED displays with high brightness and contrast. Due to repeated chip transfer and the requirement of high alignment, the manufacturing and commercialization of a micro-LED display utilizing pick-and-place technology will suffer serious accuracy problems when the minimum size of RGB subpixels shrinks to 10 μm. Furthermore, due to the non-uniform wavelength distribution within a large epitaxial wafer and the substantially temperature-sensitive performance of AlGaInP-based red LEDs, the pick-and-place technology would not shine. GaN-based blue LEDs, combining green and red color converters, such as quantum dots, with blue LEDs as a pumping source is one of the most promising techniques for full-color micro-LED display manufacturing [9,15,16].

Quantum dots (QDs) are artificial nanocrystals that exhibit three-dimensional quantum confinement. The quantum confinement effect is controlled by changing the size of the quantum dot crystal, which allows the physical properties of the material to be changed and the emission wavelength of the quantum dot crystal to be modulated. Colloidal quantum dots (QDs) have been shown to have a higher quantum yield (QY), a narrower emission linewidth, and a shorter luminous lifetime [17]. As a result, recent demonstrations of QD-based RGB micro-LED arrays have identified them as promising color conversion materials [18,19]. The passivation of the surface of QD films using polymers is a well-known approach for lowering the density of surface trap states, preventing non-radiative recombination, and thereby increasing the QD’s QY [20]. Moreover, the passivation technique of atomic layer deposition (ALD) provides excellent step coverage passivation and uniformity. The ALD technique plays an important role in micrometer scale LEDs, in which it can suppress the leakage current caused by sidewall defects as well as decrease the Shockley–Read Hall (SRH) non-radiative recombination [21,22,23,24]. On the other hand, due to better dielectric quality, the ALD technique also can be used on the passivation of quantum dots, which can prevent moisture and oxidation. Despite the fact that QD passivation minimizes QY degradation, these approaches limit light absorption due to the lower volume density of an active region, which is often the core of QDs [25,26,27]. Inkjet printing is a low-cost printing technique that allows for large-area manufacturing without the use of a mask, making colloidal QDs a good choice for next-generation display technology [28]. As a result, for a QD-based micro-LED display, a novel structure that can simultaneously enhance the light absorption and stability of QDs is desired. Light-scattering media have recently been employed in energy harvesting devices such as solar and photo electrochemical cells to enhance light absorption [29,30,31]. Light undergoes multiple scattering and diffusive transport as the scattering strength and medium thickness increase [32], leading to increased optical path length and enhanced light absorption [18]. A combination of the scattering medium and GaN-based LEDs is extremely desirable, due to the commercialization and development of highly efficient GaN-based blue LEDs, as well as GaNs’ low light absorption in the visible spectrum [33]. 

In this study, we demonstrated a new type of micro-LED array based on nanoporous-GaNs (NP-GaN) embedded with QDs, as well as a novel NP-GaN structure that can scatter light and host QDs (NPQD). Moreover, the self-aggregation issue of QDs would be improved by NP-GaN embedded with QDs, resulting in significantly increased light absorption due to multiple light scattering compared to QD film structure. Furthermore, the NP-GaN structure provides a viable pathway to develop high-uniform color conversion micro-LED displays. 

## 2. Experiment and Fabrication Process

A highly efficient QDs micro-LED array with full-color emission capability was developed in this study. A heavily n-doped blue micro-LED 4 × 4 array with a 20 × 20 μm^2^ size was developed first. Undoped gallium nitride (u-GaN) was grown on a double-sided polished sapphire substrate using metal-organic chemical vapor deposition (MOCVD). After that, the n^+^-GaN (n = 5 × 10^18^ cm^−3^) was grown, followed by the growth of multiple pairs of InGaN/GaN quantum well structures, and finally p-GaN epitaxial growth to complete the micro-LED epitaxial wafer.

**Figure 1:**
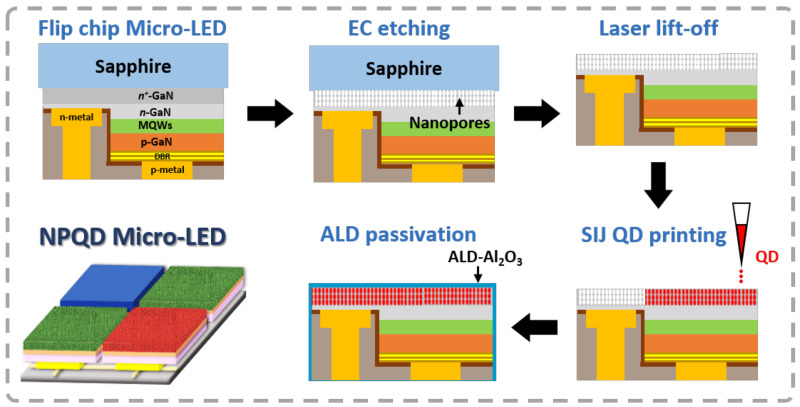
The process flow of NP-GaN embedded with QD Micro-LED device.

There were several parts to the device fabrication process, and the entire process flow for the full-color micro-LED device fabricated using NP-GaN embedded with QD is shown in Figure 1. After the fabrication of micro-LED chips, a NP-GaN structure in n^+^-GaN was made via electrochemical etching using oxalic acid (C_2_H_2_O_4_) with a molarity of 0.3 M at room temperature. Next, the sapphire substrate of the micro-LED was removed using laser lift-off technology. Then, QDs were printed in NP-GaN by using super inkjet (SIJ) printing technology to achieve full-color NP-GaN micro-LED display. SIJ is a novel type of electrohydrodynamic (EHD) printer with liquid atomization technology based on electrospraying that used an oscillating electric field to provide the necessary pressure for droplet ejection. This technique enables the formation of sub-femtoliter droplets, which corresponds to a resolution of around 1 μm on the substrate if the wetting circumstances are favorable. The strength of the electric field between the ink meniscus at the tip of the nozzle and the substrate is determined by the amount of charge applied to the charging electrode, which charges the ink inside the nozzle. If the field is strong enough, a droplet will be drawn out, and the ink will be charged by applying a pulsed voltage to the charging electrode, resulting in the formation of a droplet with each pulse [22]. Finally, low temperature (50 °C) ALD technology was performed using aluminum oxide (Al_2_O_3_) for the passivation of QDs. This passivation layer not only prevents the quantum dots from being affected by high temperature and any changes in material characteristics, but it also protects the QDs from moisture and oxidation. 

**Figure 2:**
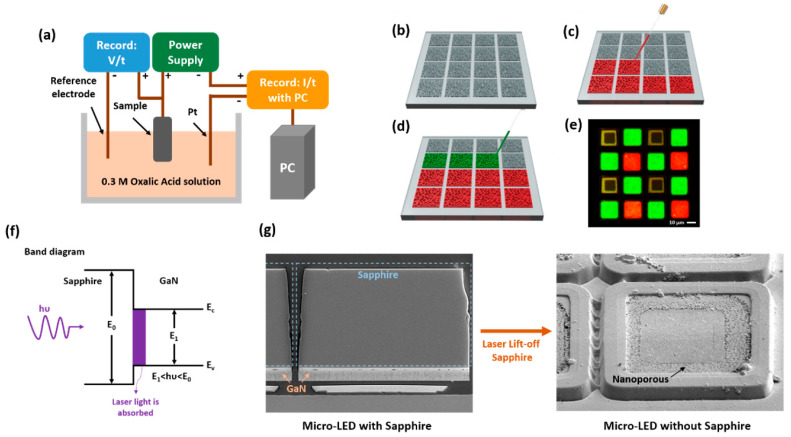
(**a**) The scheme diagram of the electrochemical etching setup; (**b**) the structure of the NP-GaN array; (**c**) red QDs and (**d**) green QDs injected to NP-GaN the array by SIJ-S050; (**e**) fluorescence optical microscopy image of RGB NP pixel; (**f**) band diagram for the laser lift-off process and (**g**) SEM image of the sapphire substrate removed by laser lift-off technology.

The electrochemical etching was carried out to form nanopores in 4.8-μm thickness on the n-GaN layer (n = 5 × 10^18^ cm^−3^), which was grown on a double side polished sapphire substrate, and the setup is schematically shown in Figure 2a. Oxalic acid (0.3 M) was used as an electrolyte at room temperature with constant magnetic stirring. The anode and cathode were made up of an indium-contacted GaN sample and a wire, respectively. The sample was exposed to a constant voltage (22 V) and the resulting current increase was measured. The electrochemical etching technique described above completed the nanopores in the n-GaN, transforming it into a light-scattering medium and a structure that could be embedded with quantum dots.

Before printing the QD in the NP-GaN structure, the sapphire substrate of the micro-LED should be removed by laser lift-off technology [34]. The high power ultraviolet pulsed laser is irradiated to the interfacial GaN from the backside of sapphire substrate. An excimer laser with a wavelength of 248 nm was used in this study to initiate the plasma and shock waves. Due to the higher absorption properties of GaN crystal over sapphire in the UV spectrum range, the laser energy selectively heats up the GaN to thermally disintegrate it, whereas the laser light passes through sapphire unaffected, as in Figure 2f. After the laser lift-off process, the compressive stress in a GaN film is relieved, which is primarily caused by the thermal match between the GaN film and the sapphire substrate, as shown in Figure 2g. The inkjet printing process of the QDs started with the preparation of a colloidal quantum dot solution to enhance light scattering by mixing with light scatters such as ZnO nanoparticles [35]. The propagation direction of the blue light traveling in QDs with the ZnO nanoparticle mixture could become randomized, increasing the absorption of pumping blue light by the QDs. Secondly, SIJ-S050 was used to load QDs onto the NP-GaN structure, with configurable parameters such as the working distance between the nozzle and the substrate, as well as the AC and DC voltages. The full-color micro display was then achieved by printing red and green QDs in NP-GaN with a 3-μm thickness with the printing process flow of QDs NP-GaN array shown in Figure 2b–d. Moreover, we used CdSe/ZnS core-shell QDs, which excited the peak wavelength of 630 and 537 nm and exhibited over 90% the PLQY of phosphor, which was purchased from Unique Materials Co., Ltd. The fluorescence optical microscopy images of the 20 × 20 μm^2^ NP micro-LED sub-pixel arrays are shown in Figure 2e.

## 3. Results and Discussion

### 3.1. Full-Color Nanoporous Quantum Dots Micro-LED Array

**Figure 3:**
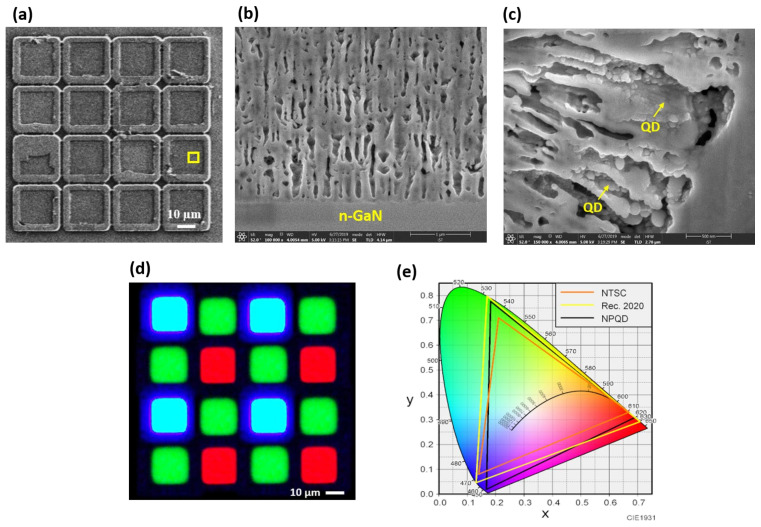
(**a**) Top view SEM of flip chip NP micro-LED array; (**b**) cross-sectional SEM of NP-GaN; (**c**) SEM image of NP-GaN structure embedded with QDs; (**d**) electroluminescence optical microscopy image, and (**e**) the CIE-1931 chromaticity diagram of RGB NP pixel micro-LED.

The NP-GaN micro-LED array with a single pixel size of 20 × 20 μm^2^, and a pitch of 25 μm was fabricated, as shown in Figure 3a. The EC etching morphology strongly depends on the anodic bias and electrical conductivity of GaN layers, and the EC etching was stopped at the n^+^-GaN/n-GaN interfaces. Due to the refractive index contrast between the air and GaN, the NP-GaN scattering light randomized the propagation directions of the incident light and increased the optical path. The scanning electron microscope (SEM) image shows the position of nanopores which were randomly distributed in n^+^-GaN, and the size of pores ranges from 100 to 500 nm, as shown in Figure 3b. Due to this light scattering structure, light propagated through it and causes the decrease of the transport mean free path (TMFP) [16]. The TMFP was defined as the average distance after which light propagation is fully randomized, from the measured transmittance.

The cross-sectional SEM images of NPQD indicated the clustered QDs, as shown in Figure 3c. To compare the QD film on the planar LED and NP-GaN structure embedded with QD, the total transmittance spectrum of the QD film and NPQDs was measured in the blue-violet region. With the wavelength decreased, the transmittance of the NPQDs significantly decreased due to the combination of the wavelength-dependent light scattering and absorption coefficients of the QDs [18]. The electroluminescence optical microscopy image of RGB NP pixel micro-LED is shown in Figure 3d. In the CIE-1931 chromaticity diagram, the overlap area of the NTSC space and the Rec. 2020 standard are 97.3% and 89.1%, respectively, as shown in Figure 3e. This significantly improved light absorption, enabling the demonstration of QD-based thin color converters with high color purity, which has been challenging due to the low absorption coefficients of green QDs.

### 3.2. Light Conversion Efficiency of NPQD

**Figure 4:**
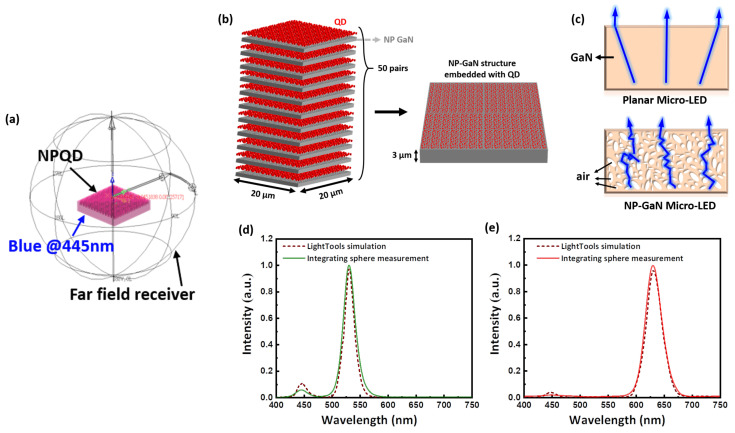
(**a**) Simulation modeling for the NP-GaN structure; (**b**) scheme diagram of an NPQD micro-LED in the SOLIDWORK model; (**c**) illustration ray tracing diagram of multiple light scattering for planar and NP-GaN micro-LED structures and the electroluminescence spectra of the measurement and simulation for (**d**) the green QD film and the green NPQD, and (**e**) the red QD film and the red NPQD.

The modeling section began with the construction of a cuboid with a thickness of 0.3 μm by LightTools Illumination Design Software. The surface of the cuboid was drilled with nano-holes of different sizes and randomly distributed. Then set amount of sphere-like objects were used to fill the nano-holes. To build a NP structure randomly distributed vertically and horizontally, these 50 cuboids were superimposed with randomly distributed holes and a sphere-like object to produce a cuboid with a thickness of 3 μm as shown in Figure 4b. The multiple light scattering medium was established with the schematic diagram of ray tracing, as shown in Figure 4c. This ray tracing model reveals that NP-GaN demonstrated longer optical path lengths which are is beneficial to uniformly pumping the QDs.

In additional, a 20 × 20 μm^2^ surface light source was built to excite the QD in the NP structure. It was a LED Gaussian light source with center emission wavelength of 450 nm, and the FWHM was set to 20 nm. Moreover, a surface receiver 20 μm away from the NP surface was set to receive the photons emitted by the NPQD micro-LED, and a far-field receiver was set to receive the luminescence spectrum, which was the same as the integrating sphere principle, as shown in Figure 4a. The light source was adjusted to 450 nm to simulate the light conversion efficiency of green and red QD. Then, the spectrum of the NPQD micro-LED was obtained for calculating their light conversion efficiency. Figure 4d,e illustrates that the electroluminescence spectra of measurement and simulation held a good agreement with each other. The LCE of the measurement and the simulation were 96.1% and 94.6%, respectively, for the red NPQD, and 90.3% and 89.5%, respectively for the green NPQD. This high LCE for NPQD with only 3-μm thickness of QD was due to the difference in the refractive index between the medium formed by GaN and air. Blue light incident on randomly distributed nanopores would have strong scattering, and the optical path will become longer and increase the possibility of exciting QDs.

**Figure 5:**
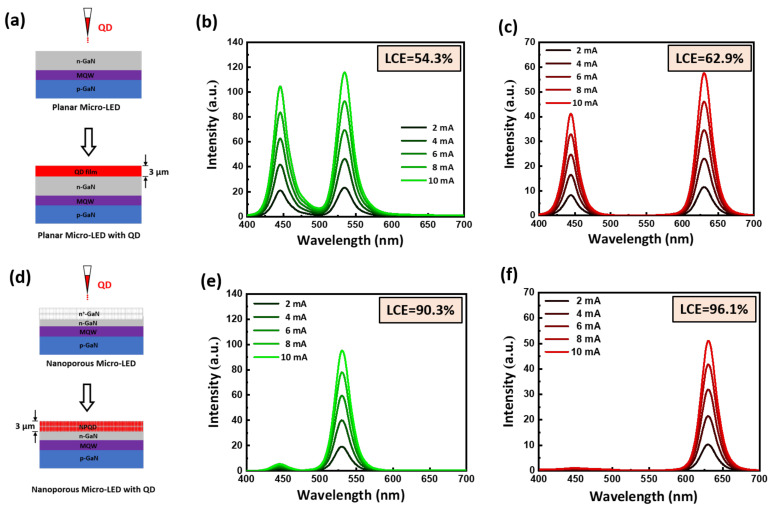
Schematic diagram of the (**a**) normal micro-LED and (**d**) nanoporous micro-LED with QDs. Electroluminescence spectrum of (**b**) the green QD film and (**e**) the green NPQD, (**c**) the red QD film and (**f**) the red NPQD with increasing applied current of blue micro-LED pumping source.

Figure 5a,d demonstrate a schematic diagram of a normal micro-LED and a nanoporous micro-LED with QDs. Furthermore, the blue emission light at 445 nm was used to excite the green and red QDs, the electroluminescence (EL) spectrum of the QD film and NPQD at various current conditions of blue micro-LED pumping source within a 2-mA interval is shown in Figure 5b,c,e,f. Due to the multiple light scattering effect, the light conversion efficiency (LCE) of the green and red NPQDs was 90.3% and 96.1%, respectively. The LCE of the NP-GaN structure was found to be enhanced by 66.2% for the green QD and by 52.7% for the red QD. This implies that the NPQDs could absorb more blue photons than the QD film and convert them to red or green photons. The NP-GaN structure provides a useful method to reduce the leakage of blue light for display, which is beneficial to the development of display techniques.

### 3.3. Illuminance Uniformity of NPQD

**Figure 6:**
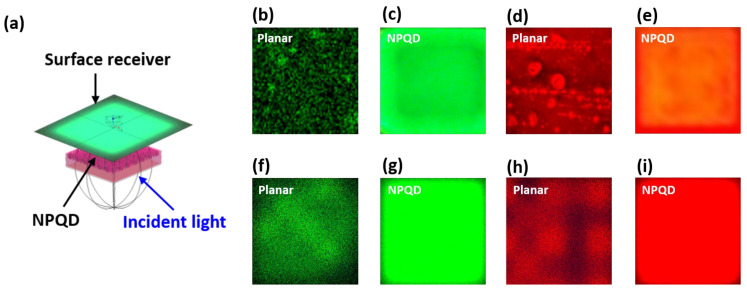
(**a**) LightTools model including surface receiver, nanoporous embedded QD structure, and light source; the measurement fluorescence optical microscopy image for planar GaN (**b**) green and (**d**) red QD, and for the NP-GaN (**c**) green, (**e**) and red structures; simulation of a luminous image in planar GaN (**f**) green, and (**h**) red QD; and with NP-GaN (**g**) green, and (**i**) red structures.

The illuminance uniformity of QD inkjet printing is poor due to the QD self-aggregation effect. The light scattering effect is amplified in our proposed nanostructure, which increases the probability of blue light stimulating the QDs. Moreover, a NP structure could maintain the position of the quantum dots to avoid the self-aggregation effect after the QD inkjet printing process, thereby improving color performance. Figure 6b–e illustrates the red and green luminous image with and without an NP structure. To analyze the color performance of the luminous image, a fluorescence luminescence optical microscope (FLOM) image was transferred to a gray level and was divided into 300 by 300 pixels by MATLAB. Illuminance uniformity was defined as a ratio between the minimum of the illuminance pixels and the average of the illuminance pixels. Green and red illuminance uniformity was found to be 54.3% and 42.9%, respectively, for the planar GaN, and 90.7% and 91.2%, respectively, for the NP-GaN. Overall, the illuminance uniformity of NPQD was about twice of QD on planar GaN. Figure 6f–i illustrates the simulation results with and without an NP-GaN structure-embedded QD. The illuminance uniformity for the red QDs in the planar GaN and the NP-GaN were 47.7% and 92.5%, respectively, and 54.5 % and 92.8 %, respectively, for the green QDs in the planar GaN and the NP-GaN. The luminous image and the illuminance uniformity have a good fitting to the measurement data shown in Table 1. As a result, the QD self-aggregation phenomenon was effectively inhibited, and high color purity was approached, which is very promising for display technology.

## 4. Conclusions

In summary, the optical properties of NP-GaN micro-LEDs embedded with QDs were demonstrated. Multiple light scattering in NP-GaN increased the optical path length of incident light as well as the opportunity of exciting QD. Colloidal QDs were loaded into the NP-GaN structure using the SIJ printing technique, resulting in 90.3% and 96.1% of LCEs for green and red within 3-μm-thick QD layers, respectively. Additionally, based on the studies on the QD inkjet printing and light absorption, green and red QDs were selectively loaded in an NP-GaN structure to carry out excellent color performance. An NP-GaN embedded with QDs mitigated the self-aggregation issue of QDs and achieved illuminance uniformity of 90.7% and 91.2% for the green and red subpixels, respectively. Finally, a wide color gamut showing 97.3% in the NTSC space and 89.1% in the Rec. 2020 standard was achieved. The high-uniform and high-efficient color conversion micro-LED using NP-GaNs embedded with quantum dots holds great promise for future displays.

## Figures and Tables

**Table 1:** Illuminance uniformity of QD film and NPQD.

Illuminance Uniformity	Green QD Film	Green NPQD	Red QD Film	Red NPQD
Measurement	54.3%	90.7%	42.9%	91.2%
Simulation	54.5%	92.8%	47.7%	92.5%

## Data Availability

The data presented in this study are available on request from the corresponding author.
