# Correction: Huang et al. High-Uniform and High-Efficient Color Conversion Nanoporous GaN-Based Micro-LED Display with Embedded Quantum Dots. *Nanomaterials* 2021, *11*, 2696

**DOI:** 10.3390/nano12020252

**Published:** 2022-01-14

**Authors:** Yu-Ming Huang, Jo-Hsiang Chen, Yu-Hau Liou, Konthoujam James Singh, Wei-Cheng Tsai, Jung Han, Chun-Jung Lin, Tsung-Sheng Kao, Chien-Chung Lin, Shih-Chen Chen, Hao-Chung Kuo

**Affiliations:** 1 Department of Photonics, Institute of Electro-Optical Engineering, National Yang Ming Chiao Tung University, Hsinchu 30010, Taiwan; s101328035@gmail.com (Y.-M.H.); kingko56@yahoo.com.tw (J.-H.C.); randyliouyuhau@gmail.com (Y.-H.L.); jamesk231996@gmail.com (K.J.S.); jj510382.ee10@nycu.edu.tw (W.-C.T.); 18618135355@flexpn.com (C.-J.L.); tskao@nycu.edu.tw (T.-S.K.); 2 Institute of Photonic System, National Yang Ming Chiao Tung University, Tainan 71150, Taiwan; 3 Semiconductor Research Center, Hon Hai Research Institute, Taipei 11492, Taiwan; 4 Department of Electrical Engineering, Yale University, New Haven, CT 06520, USA; jung.han@yale.edu; 5 Graduate Institute of Photonics and Optoelectronics, National Taiwan University, Taipei 10617, Taiwan

The authors wish to make following corrections in this paper [1].

## Authorship Correction

Shih-Chen Chen is included as a corresponding author in the updated publication. 

## Text Correction

There was an error in the original publication. In the first paragraph of page 7, in the sentence “The LCE of NP-GaN structure is found to be enhanced by 66.2% for green QD and 124.0% for red QD”, the 124.0% should be changed to 52.7%. 

In Figure 5c,f, the LCE of the red QD film and red NPQD are 62.9% and 96.1%. As a result, the enhancement is 52.7%, not 124.0%.

This change does not affect the scientific results or conclusions in the original published paper.

The authors would like to apologize for any inconvenience caused to the reader by making this change.
